# Curcumin as a double-edged sword for stem cells: dose, time and cell type-specific responses to curcumin

**DOI:** 10.1186/s40199-015-0115-8

**Published:** 2015-06-12

**Authors:** Fatemeh Attari, Maryam Zahmatkesh, Hadi Aligholi, Shahram Ejtemaei Mehr, Mohammad Sharifzadeh, Ali Gorji, Tahmineh Mokhtari, Mojtaba Khaksarian, Gholamreza Hassanzadeh

**Affiliations:** Department of Neuroscience, School of Advanced Technologies in medicine, Tehran University of Medical Sciences, Tehran, Iran; Department of Anatomy, School of Medicine, Tehran University of Medical Sciences, Tehran, Iran; Shefa Neuroscience Research Center, Khatamolanbia Hospital, Tehran, Iran; Department of Pharmacology, School of Medicine, Tehran University of Medical Sciences, Tehran, Iran; Faculty of Pharmacy, and Pharmaceutical Sciences Research Center, Tehran University of Medical Sciences, Tehran, Iran; Department of Physiology, Medical College, Lorestan University of Medical Sciences, Khorramabad, Iran; Epilepsy Research Center, WestfälischeWilhelms-UniversitätMünster, Münster, Germany

**Keywords:** Curcumin, Bone marrow mesenchymal stem cells, Neural stem cells, Cell proliferation, Cell survival

## Abstract

**Background:**

The beneficial effects of curcumin which includes its antioxidant, anti-inflammatory and cancer chemo-preventive properties have been identified. Little information is available regarding the optimal dose and treatment periods of curcumin on the proliferation rate of different sources of stem cells.

**Methods:**

In this study, the effect of various concentrations of curcumin on the survival and proliferation of two types of outstanding stem cells which includes bone marrow stem cells (BMSCs) and adult rat neural stem/progenitor cells (NS/PCs) at different time points was investigated. BMSCs were isolated from bilateral femora and tibias of adult Wistar rats. NS/PCs were obtained from subventricular zone of adult Wistar rat brain. The curcumin (0.1, 0.5, 1, 5 and 10 μM/L) was added into a culture medium for 48 or 72 h. Fluorescent density of 5-bromo-2′-deoxyuridine (Brdu)-positive cells was considered as proliferation index. In addition, cell viability was assessed by MTT assay.

**Results:**

Treatment of BMSCs with curcumin after 48 h, increased cell survival and proliferation in a dose-dependent manner. However, it had no effect on NSCs proliferation except a toxic effect in the concentration of 10 μM of curcumin. After a 72 h treatment period, BMSCs and NS/PCs survived and proliferated with low doses of curcumin. However, high doses of curcumin administered for 72 h showed toxic effects on both stem cells.

**Conclusions:**

These findings suggest that curcumin survival and proliferative effects depend on its concentration, treatment period and the type of stem cells. Appropriate application of these results may be helpful in the outcome of combination therapy of stem cells and curcumin.

## Background

Curcumin (diferuloylmethane) is one of the active components of dietary spice turmeric (Curcuma longa Linn) which was first chemically characterized in 1910 [1, 2]. To date, a great number of studies have focused on the multifarious biological effects of curcumin including its antioxidant, anti-inflammatory and cancer chemo-preventive properties [3, 4]. In addition, some papers have reported that curcumin can decrease oxidative damage and improve cognitive deficiencies related to aging. Moreover, curcumin can also be useful for the treatment of neurodegenerative diseases such as Alzheimer’s disease and brain ischemia [5, 6]. Furthermore, there are some reports on the synergistic effect of curcumin in conjunction with stem cell therapy vis-à-vis recovery from spinal cord injury [7]. In this regard, curcumin may enhance proliferation of stem cells for swift regeneration [7, 8]. Previous investigations have reported that curcumin has biphasic effects on the proliferation of some stem cells including spinal cord neural progenitor cells [9], embryonic neural progenitor cells [8] and 3 T3-L1 preadipocytes [10]. To find the optimal curcumin concentration as well as the administration time for treatment of different types of stem cells with curcumin, more studies are needed.

Among different sources of stem cells, bone marrow mesenchymal stem cells (BMSCs) are known to have capacity for proliferation and differentiation into mesenchymal and non-mesenchymal lineages. In many previous study, these types of stem cells were used for cell and gene therapy due to their capacity for self-renewal in a number of non-hematopoietic tissues as well as their multi-potentiality for differentiation [11].

On the other hand, adult neural stem/progenitor cells (NS/PCs) are self-renewal and multi-potent cells located specially in the subventricular zone of the lateral wall of the lateral ventricle of an adult mammalian brain. These cells can be isolated and cultured in-vitro to produce true neural and glial cells and finally transplanted for the treatment of neurodegenerative diseases [12, 13]. Little is known about the effective dose and treatment periods of curcumin when it is utilized as a supportive element for BMSCs and NS/PCs. Hence, in this study, the effects of various concentrations of curcumin on survival and proliferation of BMSCs and NS/PCs at different time points were investigated.

## Methods

### BMSCs culture

BMSCs were extracted from the bone marrow of bilateral femora and tibias of 4 weeks old male Wistar rats. The cell suspension was centrifuged and plated on T-25 plastic flasks in Dulbecco’s Modified Eagle Medium (DMEM/F12) (Invitrogen, USA), supplemented with 10 % fetal bovine serum (FBS, Invitrogen, USA), 100U/ml penicillin and 100 mg/ml streptomycin (Invitrogen, USA) and incubated at 37 °C with 5 % CO_2_. When primary cultures became almost confluent, the cells were passaged by trypsinization and cultured in the above compound. All of the experiments were performed using cells at passages 10-18 [14].

### NS/PCs culture

SVZ specimens were harvested from the brain of young adult male Wistar rats (150-200 g) and transported in 10 % penicillin-streptomycin solution (prepared with 0.1 M cold PBS, PH 7.2-7.4). Next, 500 μl of 0.05 % trypsin/ EDTA solution (Invitrogen, USA, 5 min at 37 °C) was used for tissue dissection. The reaction was brought to a halt by the addition of 500 μl of Soybean trypsin inhibitor (Sigma, USA) to the dissociated tissue. After centrifugation, the cells were placed on T-25 plastic flasks in 5 mL of DMEM/F12 containing 1 % N2 supplement (Invitrogen, USA), 3 % B27 supplement (Invitrogen, USA), 2 μg/mL heparin (Sigma, USA), 1 % glutamax (Invitrogen, USA), 1 % penicillin/streptomycin (Invitrogen, USA), 10 ng/ml basic fibroblast growth factor (bFGF; Millipore, Germany), 20 ng/ml epidermal growth factor (EGF; Miltenybiotech, Germany), at 37 °C in a humidified atmosphere with 5 % CO_2_. Neurospheres were passaged by trypsinization and mechanically separated after 15 days [12, 15]. The NS/PCs obtained from the third passage were used for all experiments.

### Cell viability assay

Cell viability was assessed by MTT (3-(4,5-dimethylthiazolyl-2) -2,5-diphenyltetrazolium bromide) assay. NS/PCs and BMSCs were seeded at a density of 1×10^4^ cells in 96-well plates. Curcumin (Sigma, USA) was dissolved in Dimethyl Sulphoxide (DMSO) (Sigma, USA) and added in different concentrations of 0.1, 0.5, 1, 5 and 10 μM/L into the culture medium. In the control group, cells were treated with the DMSO. Forty-eight and seventy two hours after the treatment, the medium containing curcumin was replaced with fresh medium containing 1 mg/ml MTT solution in 0.01 M PBS. The plates were incubated at a temperature of 37 °C for 4 h. The mitochondrial dehydrogenase of viable cells broke down MTT and produced purple formazan. The cells were disrupted in DMSO. The purple formazan dye was then measured with ELISA reader at 595 nm absorbance [16]. Each experiment was repeated three times.

### Immunofluorescent assay

The cells were fixed with 4 % paraformaldehyde in PBS for 1 h then rinsed with PBS three times for 5 min and incubated with blocker solution (5 % normal goat serum and 1 % bovine serum albumin in PBS) for 60 min. Mouse anti-Nestin (1:100; Abcam, USA) [12], rabbit anti-CD73 (1:200; Abcam, USA) or rabbit anti-CD105 (1:100; Abcam, USA) [17] were used at 4 °C over night. After washing with PBS, goat anti-Mouse IgG (alexa flour 647, 1:600; invitrogen, USA) or goat anti-Rabbit IgG (FITC; 1:700; Abcam, USA) were added to the cells for 60 min at room temperature and the nuclei were stained with 4,6-diamidino-2-phenylindole (DAPI, 1 g/ml, Santa Cruz, Germany) or Propidium Iodide (PI; invitrogen, USA). Immuno-labeled cells were assessed by a fluorescent microscope (Olympus, japan). In control samples, the primary antibodies were eliminated in a reaction in which no immuno-reactivity was detected.

### Bromodeoxyuridine (BrdU) Immunocytochemistry

Cells were plated onto polyornithine (50 μg/ml) coated 96-well plates at a density of 1×10^4^ cells. Cells were treated with either DMSO or curcumin at two time points (48 and 72 h). Adherent cells and neurospheres were then fixed in 4 % paraformaldehyde for 30 min. The cells were washed with 0.2 % Triton-X 100 in 0.1 M PBS (pH 7.4) three times for 5 min. DNA was denatured by exposing the cells to acid (2 M HCl, 30 min at 37 °C). After that, borate buffer (0.1 M) was added to the cells for 12 min at room temperature. Then, 5 % normal goat serum in 0.1 M PBS (pH 7.4) was added to block endogenous peroxidases. Next, the cells were incubated overnight with mouse anti-BrdU antibody (1:1000, Sigma, Germany) and thereafter exposed to FITC conjugate anti-mouse IgG (1:1000, Sigma, Germany) for BMSCs or Alexa-Fluor 647-conjugated anti-mouse IgG (1:500, Abcam USA) for NS/PCs for 2 h at room temperature. Each experiment was repeated three times.

Proliferating cells and neurospheres were incubated by 10 μM/l BrdU for 48 h and analyzed by immuno-staining [18]. Fluorescent density of Brdu positive cells were measured using image J software [19, 20]. The study had the endorsement of the ethical committee of Tehran University of Medical Sciences.

### Statistical analysis

The statistical significance was determined using the *t*-test and One-Way Analysis of Variance (ANOVA) and a Tukey post-hoc test. The results are expressed as mean ± SEM. The null hypothesis was rejected at the 0.05 level of significance.

## Results

### Isolation of BMSCs and NS/PCs

**Fig. 1:**
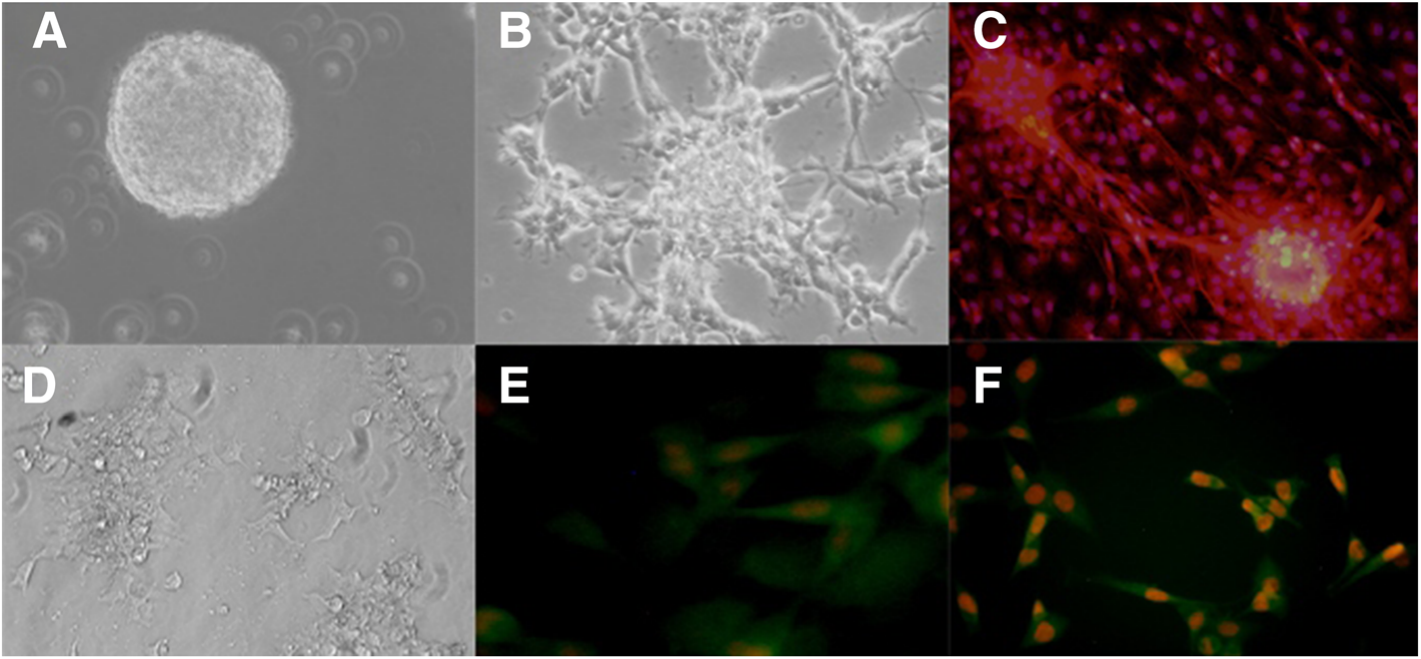
Primary culture of NS/PCs and BMSCs. NS/PCs were cultured as flouting neurospheres (**a**) or slightly adherent cells (**b**) which expressed neural stem cell marker nestin (**c**). BMSCs were cultured as adherent cells (**d**) which expressed mesenchymal stem cell markers CD105 (**e**) and CD73 (**f**)

After primary culture, BMSCs were obtained as monolayer cells which expressed mesenchymal stem cell markers CD105 and CD73. Cultivation of SVZ specimen resulted in the production of neurospheres which expressed nestin as a marker of NS/PCs (Fig. 1).

### Effect of curcumin on proliferation of BMSCs

**Fig. 2:**
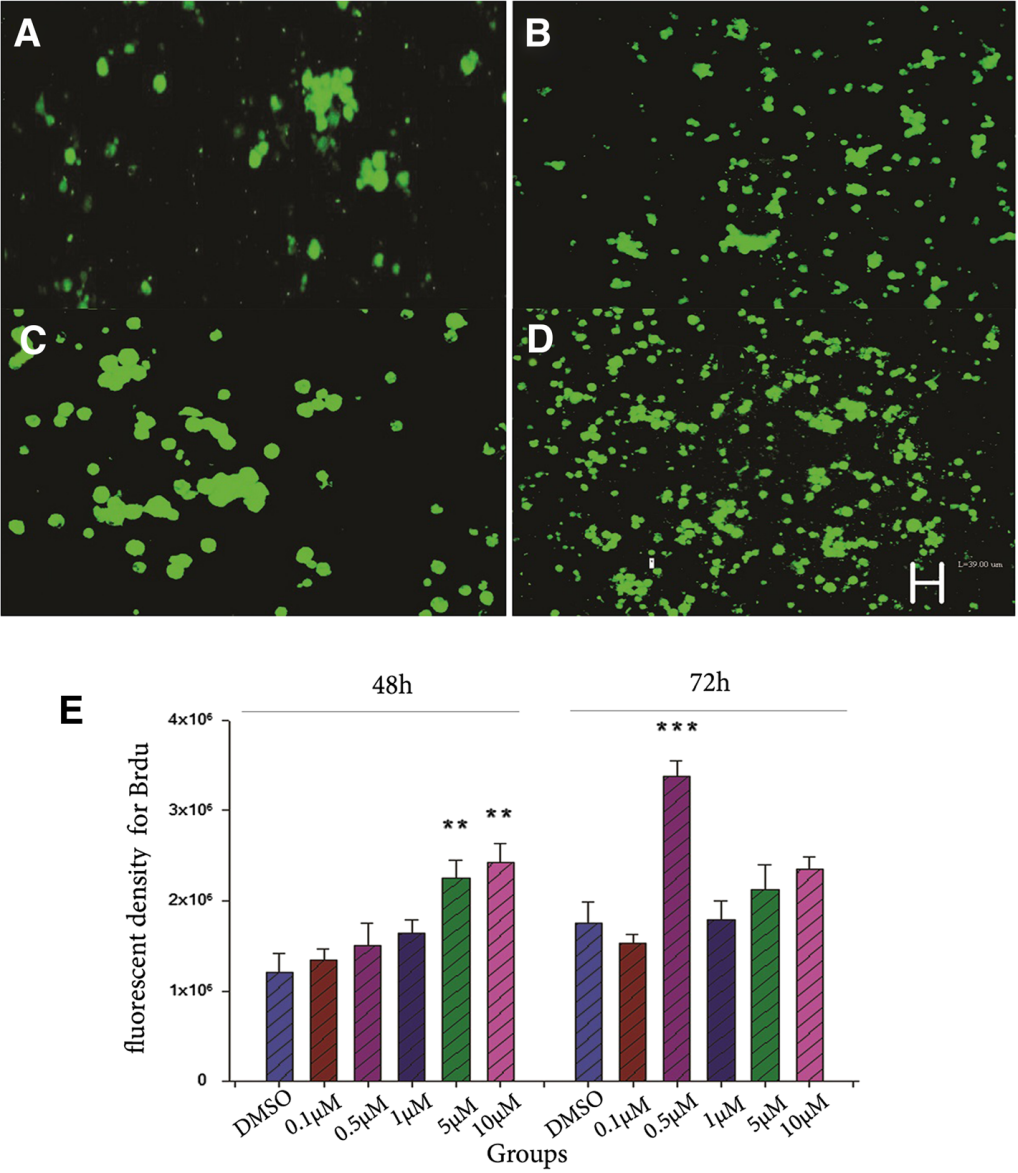
The effect of various concentrations of curcumin on proliferation of BMSCs after 48 and 72 h. Different doses of curcumin (0.1, 0.5, 1, 5 and 10 μM) added 48 or 72 h into culture medium have various proliferative effects on BMSCs . The above pictures indicate the BrdU positive cells (green) in the DMSO 48 h group (**a**), 10 μM curcumin 48 h group (**b**), DMSO 72 h group (**c**) and 0.5 μM curcumin 72 h group (**d**). Scale bare:40 μm. **e**, fluorcent density for BrdU positive cells in different doses of curcumin after 48 or 72h, **: *P* < 0.01 or ***: *P* < 0.001 vs the DMSO group

As indicated in Fig. 2, in the aftermath of 48 h administration, curcumin enhanced the BrdU positive BMSCs in a dose dependent manner, suchthat the doses of 5 and 10 μM of curcumin enhancement was statistically significant compared to the DMSO group (*P* < 0.01).

After 72 h treatment with different doses of curcumin, the above pattern was not seen, only the concentration of 0.5 μM of curcumin could considerably increase the BrdU positive BMSCs compared to the DMSO group (*P* < 0.001).

### Effect of curcumin on proliferation of NS/PCs

**Fig. 3:**
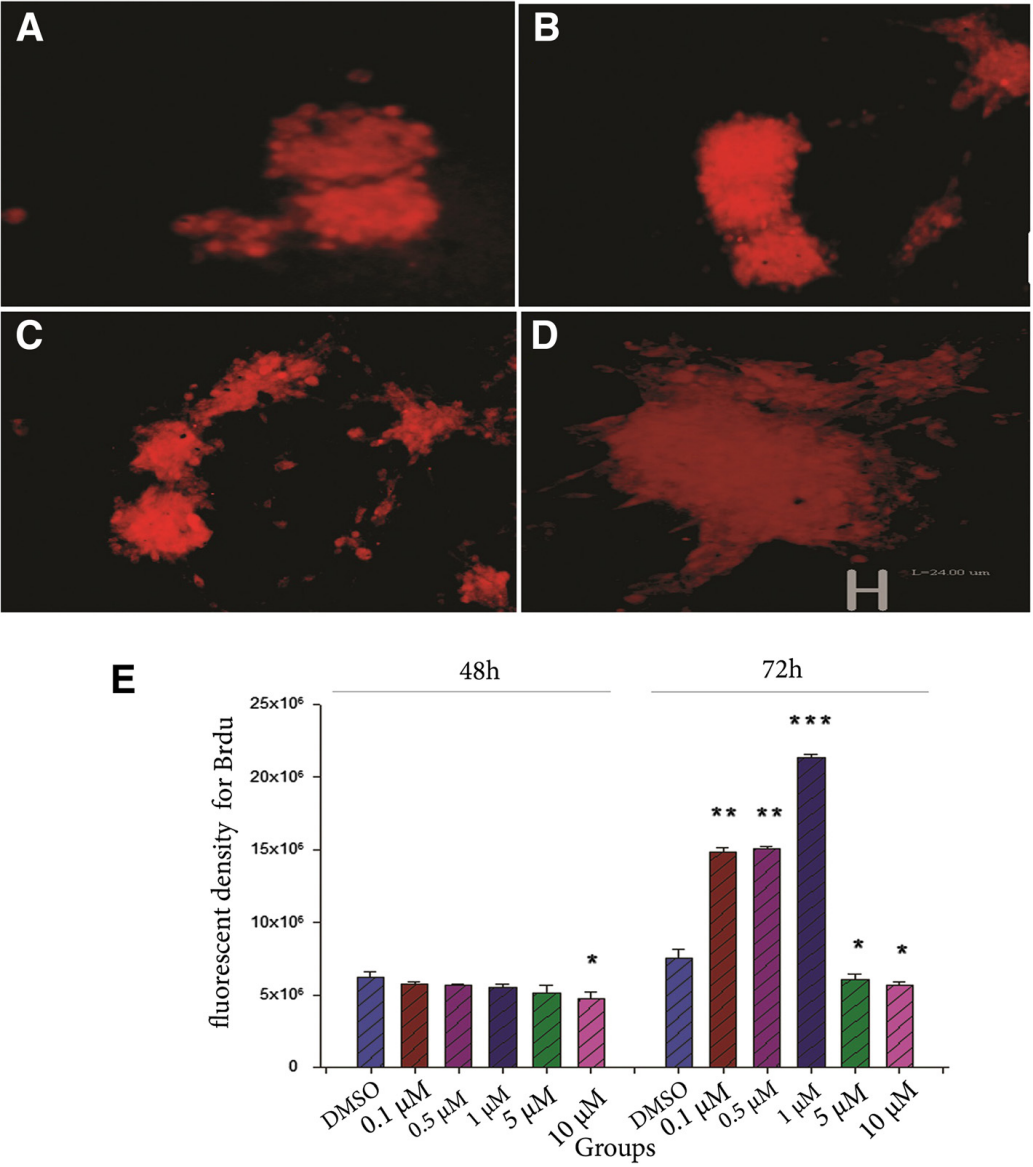
The effect of various concentrations of curcumin on proliferation of NS/PCs after 48 and 72 h. Proliferation of NSCs was evaluated using fluorescent density for Brdu-positive cells. This index was different in the groups following 48 or 72 h treatment of NS/PCs with curcumin. The above photographs show the BrdU positive cells (red) in the DMSO 48 h group (**a**), 10 μM curcumin 48 h group (**b**), DMSO 72 h group (**c**) and 1 μM curcumin 72 h group (**d**). Scale bare:24 μm. **e**, fluorcent density for BrdU positive cells in different doses of curcumin after 48 or 72h, *: *P* < 0.05, **: *P* < 0.01 or ***: *P* < 0.001 vs the DMSO group

There were no significant differences among the groups after 48 h treatment of NS/PCs with various doses of curcumin except for the 10 μM curcumin group in which the fluorescent density of BrdU was significantly decreased compared to the DMSO group (*P* < 0.05, Fig. 3).

After 72 h administration of curcumin, there was an increase in BrdU positive NS/PCs in doses of 0.1, 0.5 (*P* < 0.01) and 1 μM (*P* < 0.001) of curcumin compared to the DMSO group. The proliferation rate of NS/PCs significantly decreased following the administration of 5 or 10 μM of curcumin when compared with that of the DMSO group (*P* < 0.05, Fig. 3).

### Effect of curcumin on survival of BMSCs

**Fig. 4:**
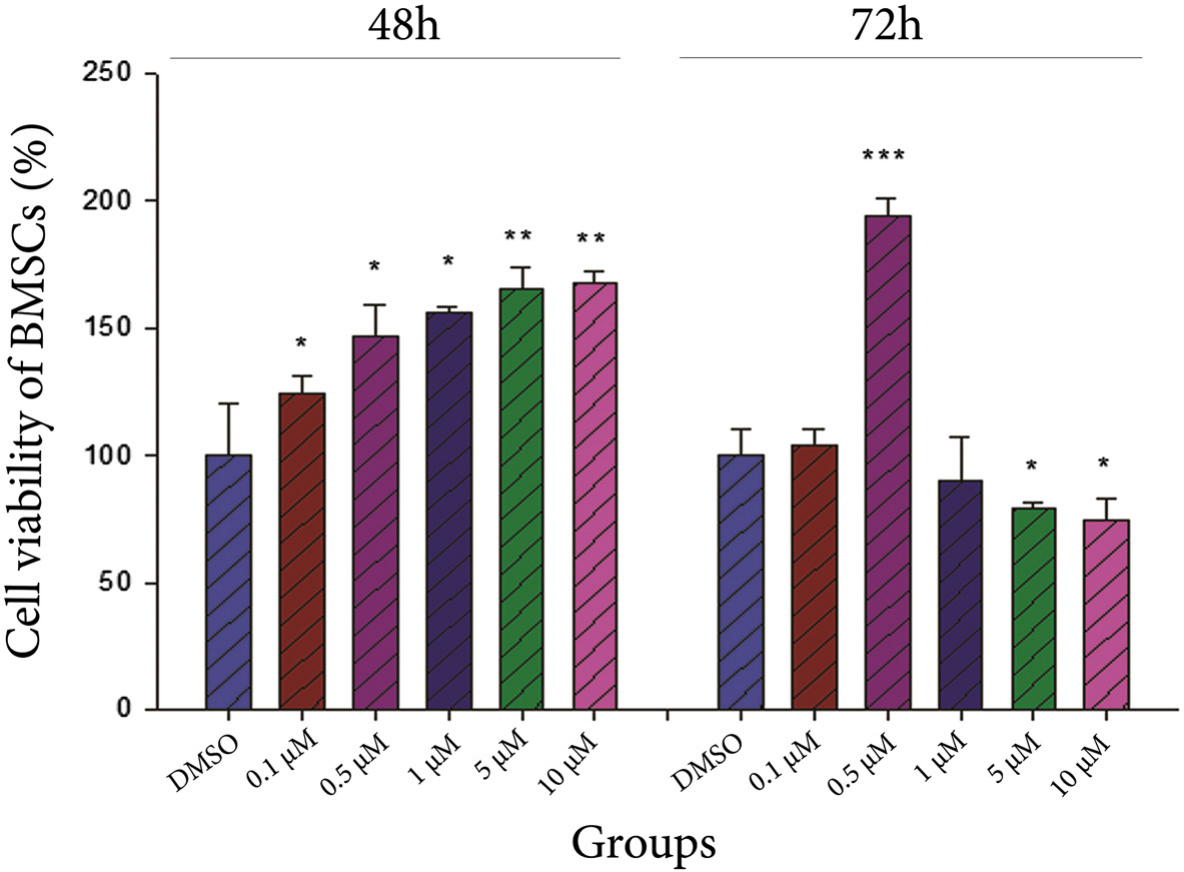
The effect of various concentrations of curcumin on viability of BMSCs after 48 and 72 h. Cell viability of BMSCs was assessed using MTT assay method. The results indicated that dose and exposure time of curcumin were the factors infuence the cell viability. *: *P* < 0.05, **: *P* < 0.01 or ***: *P* < 0.001 vs the DMSO group

The results of MTT assay showed that all concentrations of curcumin including 0.1, 0.5, 1 (*P* < 0.05), 5 and 10 μM (*P* < 0.01) could enhance the viability of BMSCs after 48 h compared to the DMSO group (Fig. 4). Following 72 h treatment, the survival of BMSCs significantly increased with 0.5 μM of curcumin (P < 0.001), but this index considerably decreased with 5 and 10 μM of curcumin (*P* < 0.05) compared to the DMSO group.

### Effect of curcumin on survival of NS/PCs

**Fig. 5:**
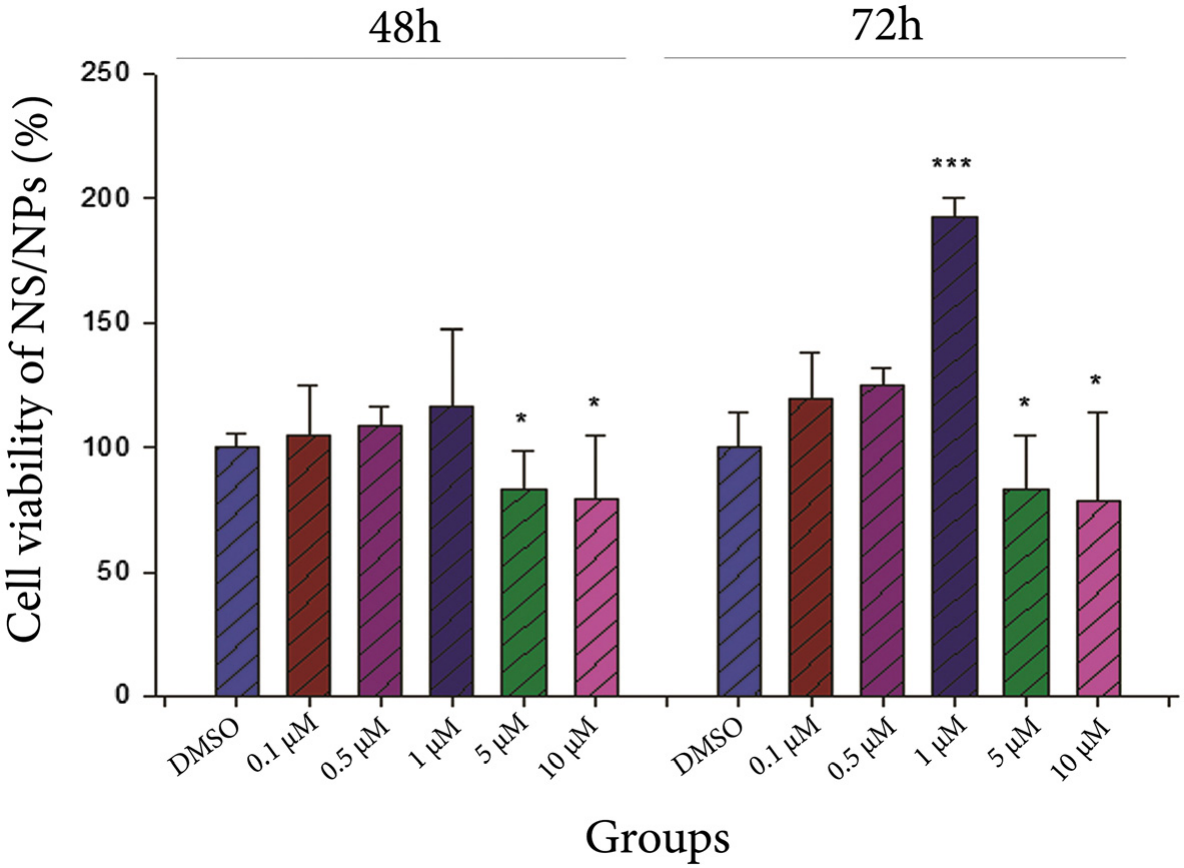
The effect of various concentrations of curcumin on viability of NS/PCs after 48 and 72 h. Curcumin in different doses had various effects on survival of NS/PCs. In addition, the cell viabilty was affected by period of treatment. *: *P* < 0.05 or ***: *P* < 0.001 vs the DMSO group

According to the results of MTT assay, curcumin with concentrations of 5 and 10 μM significantly reduced viability of NS/PCs compared to the DMSO group after 48 h (Fig. 5, *P* < 0.05). Moreover, following 72 h of NS/PCs encounter with curcumin, the highest cell viability was seen by using 1 μM of curcumin (*P* < 0.001 compared to the DMSO group). However, high doses of curcumin (5 and 10 μM) considerably decreased the viability of NS/PCs in comparison with the DMSO group (*P* < 0.05).

## Discussion

In the present study we observed multifarious effects of curcumin in terms of cell viability and stem cell proliferation. After administration of different doses of curcumin (0.1, 0.5, 1, 5 and 10 μM/L) to BMSCs, the high doses of curcumin enhanced the proliferation of BMSCs after 48 h However, when these cells were exposed to curcumin for 72 h, the increase in cell proliferation was seen only in doses of 0.5 μM of curcumin. On the other hand, the high doses of curcumin had adverse effects on the proliferation of NS/PCs while the doses of 0.1, 0.5 and 1 μM/L of curcumin administered for 72 h had beneficial effects on proliferation. The effect of curcumin on viability of BMSCs and NS/PCs was almost similar to its effect on proliferation of the cells.

Previous studies evaluated some of the effects of curcumin on the proliferation and viability of the cells. Kim et al. [8] revealed that after a 24 h treatment period, low concentration (0.5 μM) of curcumin was the most effective dose for increasing Brdu positive cells and cell viability of NS/PCs in cell culture. They opined that the stimulatory mechanism of low doses of curcumin on neural stem cells was facilitated by ERK and p38 MAP kinases [8]. In addition, Kim et al.[10] reported an increase in cell proliferation of preadipocytes with low concentrations of curcumin during a 24 h treatment period [10]. Moreover, Ormand et al. [7] reported that after 24 h, low doses of (0.5 μM) curcumin was the most effective concentration for increasing the neurosphere diameter of NS/PCs isolated from the adult rat SVZ, as a proliferation index [7]. Previous studies have shown that curcumin can be utilized at low doses to induce heme oxygenase 1 at pharmacological levels, and its induction is accompanied by generation of non-lethal levels of reactive oxygen species [3]. On the other hand, Kim et al. [8] reported a cytotoxic effect with concentrations more than 10 μM of curcumin [8]. Ormand et al. [7] also demonstrated that curcumin at higher doses caused apoptosis [7]. Another report showed that the concentrations of 1-50 μM of curcumin increased cell viability while doses higher than 80 μM decreased it [16]. The discrepancies observed among the studies may be attributed in part to the different species, duration of curcumin exposure and the dose of curcumin. In the present study, the different doses of curcumin and treatment duration were chosen based on a pilot study as well as previous reports [10, 8]. It is important to point out that according to the results of the present study; it seems that the toxic effects of curcumin on NS/PCs were more sensitive than that on BMSCs.

Indeed, in the light of latest investigations and results, curcumin acts as a double-edged sword. In other words, it can be toxic to cells and can also be helpful. It depends on the dose and type of cells. Based on this fact, curcumin should be used cautiously in future treatment strategies. Recent studies have shown that curcumin could reduce oxidative damage related to aging and was beneficial in the treatment of neurodegenerative diseases such as Alzheimer diseases, Parkinson disease, and stroke in animal models [21–25]. Xu et al. [26] showed that curcumin could reverse impaired hippocampal neurogenesis in rat model affected by chronic stress. Their results suggested that curcumin could increase the cell proliferation and neuronal populations in stress-induced behavioral abnormalities and hippocampal neuronal damage. Moreover, curcumin effects were induced by up-regulation of 5-HT_1A_ receptors and BDNF [26]. Fadhel et al. [24] indicated that administration of 200 mg/kg of curcumin by intraperitoneal method immediately, 3 h and 24 h after transient forebrain, ischemia model significantly reduced neuronal damage. This protective effect did not differ among these three different times. Fusheng et al. [21] reported that five months administration of 500 ppm curcumin in aged APP transgenic mice suppressed amyloid accumulation. Kim et al. showed that when curcumin was administered intraperitoneally at a dose of 500 nmol/kg body weight of adult mice once daily for 4 days, the Numbers of Newly Generated Cells in the Hippocampus increased [8]. However, the translation of the results of animal studies to the clinic is not an easy job. The results of phase I clinical trials indicated that curcumin is safe even at high doses (12 g/day). Humans however exhibit poor bioavailability [27]. Considering the fact that curcumin in the face of stem cells exhibits a biphasic effect, further investigation is needed to determine specific effects of curcumin on different types of stem cells.

## Conclusion

These findings suggest that curcumin proliferation effects depend on treatment period, its concentration, and the type of stem cells. These factors should be considered in stem cell therapy of degenerative and neurological disorders. Further studies are required to evaluate the synergistic effect of combination therapy of curcumin and stem cells.

## Abbreviations

BMSCs: Bone marrow mesenchymal stem cells
NS/PCs: Neural stem/progenitor cells
DMEM/F12: Dulbecco’s Modified Eagle Medium
DMSO: 3-(4, 5-dimethylthiazolyl-2)-2, Dimethyl Sulphoxide
MTT: 5-diphenyltetrazolium bromide
BrdU: Bromodeoxyuridine
ANOVA: One-Way Analysis of Variance
